# Inhibition of DYRK1A proteolysis modifies its kinase specificity and rescues Alzheimer phenotype in APP/PS1 mice

**DOI:** 10.1186/s40478-019-0678-6

**Published:** 2019-03-18

**Authors:** Benoît Souchet, Mickael Audrain, Jean Marie Billard, Julien Dairou, Romain Fol, Nicola Salvatore Orefice, Satoru Tada, Yuchen Gu, Gaelle Dufayet-Chaffaud, Emmanuelle Limanton, François Carreaux, Jean-Pierre Bazureau, Sandro Alves, Laurent Meijer, Nathalie Janel, Jérôme Braudeau, Nathalie Cartier

**Affiliations:** 1 INSERM UMR1169, 92265 Fontenay-aux-Roses, France; 2 Université Paris Saclay, Saclay, France; 3 INSERM UMR894, Centre de Psychiatrie et Neurosciences, Université Paris Descartes, Sorbonne Paris Cité, Paris, France; 4 UMR 8601 CNRS, Laboratoire de Chimie et Biochimie Pharmacologiques et Toxicologiques, Université Paris Descartes-Sorbonne Paris Cité, 75270 Paris, France; 5 Laboratoire Sciences Chimique de Rennes, UMR CNRS 6226, Groupe ICMV, Université de Rennes 1, 35042 Rennes, France; 6 ManRos Therapeutics, Hôtel de Recherche, Centre de Perharidy, 29680 Roscoff, France; 7 Sorbonne Paris Cité, Adaptive Functional Biology, Université Paris-Diderot, UMR CNRS, 8251 Paris, France; 8 CEA, DRF Institut François Jacob, MIRCen, 92265 Fontenay-aux-Roses, France; 9 Institute for Brain and Spine (ICM) Hôpital Pitié –Salpêtrière, Université Paris Sorbonne, 47 boulevard de l’Hôpital 75013, Paris, France

**Keywords:** Alzheimer’s disease, DYRK1A, Proteolysis, Kinase specificity, Therapeutic approach

## Abstract

Recent evidences suggest the involvement of DYRK1A (dual specificity tyrosine phosphorylation-regulated kinase 1 A) in Alzheimer’s disease (AD). Here we showed that DYRK1A undergoes a proteolytic processing in AD patients hippocampus without consequences on its kinase activity. Resulting truncated forms accumulate in astrocytes and exhibit increased affinity towards STAT3ɑ, a regulator of inflammatory process. These findings were confirmed in APP/PS1 mice, an amyloid model of AD, suggesting that this DYRK1A cleavage is a consequence of the amyloid pathology. We identified in vitro the Leucettine L41 as a compound able to prevent DYRK1A proteolysis in both human and mouse protein extracts. We then showed that intraperitoneal injections of L41 in aged APP/PS1 mice inhibit STAT3ɑ phosphorylation and reduce pro-inflammatory cytokines levels (IL1- β, TNF-ɑ and IL-12) associated to an increased microglial recruitment around amyloid plaques and decreased amyloid-β plaque burden. Importantly, L41 treatment improved synaptic plasticity and rescued memory functions in APP/PS1 mice. Collectively, our results suggest that DYRK1A may contribute to AD pathology through its proteolytic process, reducing its kinase specificity. Further evaluation of inhibitors of DYRK1A truncation promises a new therapeutic approach for AD.

**Electronic supplementary material:**

The online version of this article (10.1186/s40478-019-0678-6) contains supplementary material, which is available to authorized users.

## Introduction

Despite considerable academic and pharmaceutical investments during the last three decades, there is still no effective treatment for Alzheimer’s disease (AD). AD neuropathology is mostly defined by the accumulation of amyloid-beta (Aβ) plaques and neurofibrillary tangles (NFTs) in patient brains. Extracellular Aβ plaques are mainly formed by the aggregation of amyloid peptides (Aβ_40_ and Aβ_42_) whose production and accumulation are key elements in AD development [15]. Intracellular NFTs are composed of aggregated hyper- and abnormal phosphorylated Tau proteins. Accumulating data from preclinical and clinical studies have established that several immune system-mediated factors, mainly driven by glial cells, also contribute to AD pathogenesis. Astrocytes and microglia surround amyloid plaques and release cytokines leading to inflammatory processes whose dysregulation contributes to AD pathology [19, 37, 38].

Dual-specificity tyrosine-phosphorylation-regulated kinase 1A (DYRK1A), encoded by a gene localized in the Down syndrome critical region of chromosome 21, is a serine/threonine protein kinase which contributes to various biological processes in the embryonic and adult central nervous systems [39]. In AD, DYRK1A is especially known to phosphorylate Tau at several sites including Thr181, Thr212, and Thr231, which are all observed in NFTs of AD brains [29, 33, 46] and the amyloid precursor protein (APP) at Thr-668 [34] or the presenilin 1 (PS1) at Thr-354 [36]. DYRK1A also phosphorylates several immune response mediators associated with AD, including calcineurin-nuclear factor of activated T cells (NFAT) [1] and signal transducer and activator of transcription-3 (STAT3) [26]. Recent study showed that DYRK1A inhibition reduces APP phosphorylation and insoluble Tau phosphorylation and thereby reverse cognitive deficits in AD mice [3]. However, previous contradictory studies have been published [11, 23] and further study are required to confirm the DYRK1A protein levels in brain of individuals with AD. Furthermore, it is emphasized that level of its kinase activity is still unknown. Thus, the relevance of inhibiting kinase activity of DYRK1A in AD remains a matter of debate.

Here, we show that DYRK1A is truncated in the AD context. This increase of truncated forms of DYRK1A (DYRK1A_*T*_) is associated with a decrease of full-length form of DYRK1A (DYRK1A_*FL*_) thus confirming previous report by Jin and colleagues [23]. This was observed in hippocampus from AD patients but also in APP/PS1 mice, an amyloid mouse model of AD [22]. We demonstrated for the first time that this proteolysis is occurring in astrocytes and is not associated with a modification of the global DYRK1A kinase activity in AD. In vitro, we show that, compared to DYRK1A_*FL*_, DYRK1A_*T*_ exhibit stronger affinity toward STAT3ɑ. We identified Leucettine L41, derived from the marine sponge alkaloid Leucettamine B [8, 41], as an appropriate compound to inhibit DYRK1A proteolysis. To decipher the effects of DYRK1A proteolysis and its inhibition in vivo, we treated APP/PS1 mice with the leucettine L41. We show in the present study that L41 prevents DYRK1A proteolysis and reduces STAT3ɑ phosphorylation in APP/PS1 mice. Neuroinflammation, amyloid plaque load, synaptic plasticity and cognitive functions are improved. Altogether, our results confirm the involvement of DYRK1A_*T*_ in AD pathology and demonstrate the relevance of inhibitors of DYRK1A cleavage as a potentially relevant therapeutic strategy.

## Material and methods

### Animals

Fourteen APP*swe*/PS1*ΔE9* mice (referred to as APP/PS1; Jackson Laboratories) and 12 age-matched littermate control mice were used for behavioral (Morris Water Maze), pathology, and biochemistry studies. A second cohort composed of 14 APP/PS1 and nine littermates were used for behavioral (Y-maze) and electrophysiological analysis. APP/PS1 mice express the human APP gene carrying the *Swedish* double mutation (K595 N/M596 L). In addition, they express the human PS1∆E9 variant lacking exon 9 [22]. Only male mice were used. The ages at treatment and analysis/sacrifice are given in the Results section. All experiments were conducted in accordance with the ethical standards of French, German, and European regulations (European Communities Council Directive of 24 November 1986). The supervisor of in vivo studies (J Braudeau) received official authorization from the French Ministry of Agriculture to carry out research and experimentation on animals (authorization number APAFIS#4449–2,016,031,012,491,697).

### Tissue collection and sample preparation

Mice were anesthetized with ketamine/xylazine (100 and 10 mg/kg respectively) and decapitated. One hemisphere was post-fixed by incubation for 72 h in 4% PFA, cryoprotected in 30% sucrose in PBS, and cut into 40 μm sections with a freezing microtome (Leica) for histological analyses. The contralateral hemisphere was dissected for hippocampus isolation. Samples were homogenized in a lysis buffer (150 mM NaCl and 1% Triton in Tris-buffered saline) containing phosphatase (Pierce) and protease (Roche) inhibitors and centrifuged for 20 min at 15000 x g. The same procedure was applied to human samples.

### Leucettine L41 treatment

The pre-weighed compound was dissolved in DMSO/PEG300/water (5/35/60) to a final concentration of 2 mg/mL for a dose of 20 mg/kg. The formulation was prepared on the day of the in vivo experiment. The mice received five intraperitoneal injections per week for four weeks.

### DYRK1A in vitro proteolysis

Human hippocampus tissue and 4 months-aged mouse (C57Bl6) hippocampus were homogenized in 9 volumes of buffer consisting of 50 mM Tris-HCl (pH 7.4), 8.5% sucrose, 10 mM β-mercaptoethanol, 2.0 mM EDTA, followed by centrifugation at 16,000×g at 4 °C for 10 min. The supernatants were incubated in the presence or absence of various concentrations of Ca^2+^ with or without Harmine, Leucettine LeuI or Leucettine L41 at various concentrations (0.1; 1.0 or 2.0 μM) for 10 min at 30 °C. The reactions were terminated by the addition of 4-fold concentrated SDS-PAGE sample buffer, followed by heating in boiling water for 5 min. The products of proteolysis were analyzed by Western blots developed with antibody to DYRK1A.

### Identification of DYRK1A interactions

Homogenized total proteins from mouse hippocampus tissue were incubated with 2 mM EDTA, 0 or 2 mM of Ca2+ and 0 or 1 μM of Leucettine L41 during 10 min at 30 °C. 200 μg of total proteins were incubated with 2 μg of *α-DYRK1A-Nter* antibody (DYRK1A D1694) overnight at 4 °C. The proteins interacting with DYRK1A were revealed by Western blots developed with STAT3 (1/1000, Cell Signaling), NFATc1 (1/1000, Cell Signaling), APP, Tau and PS1.

### Western blotting

Equal amounts of protein (30 μg) were separated by electrophoresis in NuPAGE Bis-Tris Gels (Life Technologies) and transferred to nitrocellulose membranes. The membranes were hybridized with various primary antibodies (DYRK1A 7D10 (1/250, Abnova), DYRK1A D1694 (1/500, Sigma), GFAP (DAKO, 1/4000), Vimentin (1/1000, Abcam), pSTAT3(Y705) (1/1000, Cell Signaling), STAT3 (1/1000, Cell Signaling), IBA1 (1/2000, Wako), CD68 (1/1000, BioLegend), IDE (1/200, Santa Cruz), TREM2 (R&D Systems, 1/500), GAPDH (1/1000, Abcam)). Various secondary antibodies were also used (ECL Anti-mouse horseradish peroxidase linked, 1/2000, GE Healthcare; ECL Anti-rabbit horseradish peroxidase linked, 1/2000, GE Healthcare). Membranes were developed using enhanced chemiluminescence (Thermo Fisher Scientific). Signals were detected with Fusion FX7 (Vilber Lourmat) and analyzed and quantified using ImageJ (NIH).

### Elisa

Inflammatory cytokines and interleukin concentrations were measured using the MSD Mouse V-PLEX Plus Proinflammatory Panel 1 kit (Meso Scale Diagnostics). Extracted soluble Aβ forms were quantified with the MSD Human and rodents V-Plex Plus Aβ Peptide Panel 1 (4G8) (Meso Scale Diagnostics). All ELISA were performed according to each kit manufacturer’s instructions.

### Calpain activity

Calpain activity was measured using the fluorogenic peptide N-Succinyl-Leu-Tyr-7-Amido-4-Methylcoumarin as described by Kohli et al. [25]. Briefly, 60 μg brain extract in a final volume of 40 μL was added to 160 μL 50 μM N-Succinyl-Leu-Tyr-7-Amido-4-Methylcoumarin dissolved in dimethyl sulfoxide and Tris buffer (100 mM Tris-HCl, 145 mM NaCl at pH 7.3). Proteolysis of the substrate was monitored for 21 min at room temperature with a FlexStation3 multi-mode microplate reader (excitation: 380 nm, emission: 460 nm; Molecular Devices) either in the presence of 10 mM Ca^2+^ or 10 mM EGTA to determine calcium-independent activity, thus excluding cathepsin activity.

### DYRK1A kinase activity assay

Catalytic activity of total and endogenous DYRK1A was assessed using high-performance liquid chromatography (HPLC), based on the separation and quantification of specific fluorescent peptides (substrate and phosphorylated product), as previously described [4]. Both negative (without protein) and positive controls (DYRK1A recombinant protein) have been used to determine background and to check the specificity of the kinase activity assay.

### Immunohistochemistry and image acquisition

Cryosections were washed with 0.25% Triton in 0.1 M PBS, incubated in an 88% formic acid solution for 15 min (antigen retrieval), and saturated by incubation in 0.25% Triton in 0.1 M PBS/5% goat serum. They were then incubated with primary antibodies (DYRK1A 7D10 (1/100, Abnova), DYRK1A D1694 (1/100, Sigma), GFAP (DAKO, 1/2000), APP 4G8-Biotin, (1/1000, Covance), IBA1 (1/500, Wako)). For non-fluorescent immunostaining, endogenous peroxidase was quenched with PBS containing 3% H_2_O_2_ for 5 min, followed by amplification using the ABC system (VECTASTAIN Elite ABC HRP Kit, Vector Laboratories, Burlingame, CA, USA). Horseradish peroxidase conjugate and 3,3′-diaminobenzidine were then used according to the manufacturer’s manual (Vector® DAB, Vector Laboratories, Burlingame, CA, USA). The sections were mounted, dehydrated by passing twice through ethanol and toluol solutions, and cover-slipped with Eukitt (O. Kindler). Images were captured with a Leica DM6000 microscope and analyzed using ImageJ software (NIH). For fluorescent immunostainings, slices were incubated with secondary Alexa Fluor conjugated antibodies (Invitrogen). Slices were stained with DAPI (1/5000, Sigma), mounted in Vectashield fluorescent mounting media (Vector laboratories) and conserved at 4 °C. Images were captured with a Leica SP8 confocal microscope (Leica) and analyzed using ImageJ software (NIH). Laser power, numeric gain, and magnification were kept constant between animals to avoid potential technical artefacts. Images were first converted to 8-bit gray scale and binary thresholded to highlight positive staining. At least three sections per mouse (between − 1.7 mm to − 2.3 mm caudal to Bregma) were quantified. The average value per structure was calculated for each mouse. For quantification of Iba1 and GFAP immunoreactivity around plaques, a region of interest (ROI) was drawn around the center of the plaque. The diameter of the circular ROI was set to two times the diameter of the plaque. Mean fluorescence intensity values were measured for either DYRK1A (7D10) (*α-DYRK1A-Cter*), DYRK1A (D1694) (*α-DYRK1A-Nter*) Iba1, or GFAP immunoreactivity and were processed using Icy software (Institut Pasteur, Paris, France). Experiments and data analysis were performed blind with respect to treatments and genotypes.

### Behavioral assessment

*Y-maze.* Experiments were performed in a maze consisting of three transparent plastic arms, 46 × 11 × 25 cm each, set at a 120° angle relative to each other. During the first trial, mice could freely explore two arms, called familiar arms (FA), for 3 min, whereas the third arm was blocked by an opaque door. Assignment of arms was counterbalanced randomly within each experimental group to avoid any preference-related bias. Mice were then returned to their home cage for 5 min. Finally, mice were returned to the maze and allowed to explore all three arms, including the novel arm (NA), for 3 min. The maze was carefully cleaned with a 70% ethanol solution between each exploration phase to remove any olfactory cues. EthoVision software was used for recording and analysing each exploration trial.

### Morris water maze

Experiments were performed in a tank 180 cm in diameter and 50 cm deep, filled with opaque water maintained at 21 °C, equipped with a platform of 18 cm in diameter, submerged 1 cm below the surface of the water. Visual clues were positioned around the pool and luminosity was maintained at 350 lx. The mice were initially exposed to a learning phase, which consisted of daily sessions (three trials per session) on five consecutive days. The starting position varied pseudo-randomly, between the four cardinal points. There was a mean interval of 20 min between trials. The trial was considered to have ended when the animal reached the platform. Long-term spatial memory was assessed 72 and 120 h after the last training trial in a probe trial in which the platform was no longer available. Animals were monitored with EthoVision software.

### Ex vivo electrophysiology

*Slice preparation.* Mice were anesthetized with halothane and decapitated. The brain was rapidly removed from the skull and placed in chilled (0–3 °C) artificial cerebrospinal fluid (ACSF) containing 124 mM NaCl, 3.5 mM KCl, 1.5 mM MgSO4, 2.5 mM CaCl2, 26.2 mM NaHCO3, 1.2 mM NaH2PO4, and 11 mM glucose. Transverse slices (300–400 μm thick) were cut with a vibratome and placed in ACSF in a holding chamber, at 27 °C, for at least one hour before recording. Each slice was individually transferred to a submersion-type recording chamber and submerged in ACSF continuously superfused and equilibrated with 95% O2, 5% CO2.

### Extracellular field recordings

Electrically induced long-term potentiation (LTP) was studied. Theta-burst stimulation (TBS), mimicking the natural stimulation at the theta frequency from the medial septum to the hippocampus, consisting of five trains of four 100 Hz pulses each, separated by 200 ms, was applied at the test intensity. The sequence was repeated three times, with an interburst interval of 10s. Testing with a single pulse was then performed for 60 min (TBS) or 75 min (3 × 100 Hz), to determine the level of LTP.

### Statistical analysis

Data are expressed as the mean ± SEM. Statistical analyses were performed with GraphPad Prism (GraphPad Soft-ware, La Jolla, CA, USA) software. One-way ANOVA followed Tukey’s post-hoc tests were used to determine the significance of differences between groups. Student’s t test was used when only two groups were analyzed. All values are given as mean ± SEM. Statistical significance was set to a *P*-value < 0.05 for all tests.

## Results

### DYRK1A_T_ is detected in astrocytes of Alzheimer’s disease patients

We evaluated DYRK1A proteolysis in hippocampus from AD patients (Braak V-VI, Thal IV-V) (*n* = 4) and age-matched controls (*n* = 4). Using the anti-DYRK1A antibody 7D10 (named after α-DYRK1A-Cter) targeting the C-terminal region of DYRK1A, we observed decreased levels of DYRK1A in AD patients compared to controls (*p* < 0.05) (Additional file 1: Figure 1A,B). DYRK1A can be cleaved by calpains [23], calcium-activated cysteine proteases highly activated in AD brain [27]. Here, we found that calpain activity was higher in hippocampus of AD cases compared to controls (*p* = 0.0571) (Additional file 1: Figure 1C) and significantly correlated with DYRK1A protein levels (correlation coefficient r = − 0.94, *p* < 0.0005) (Additional file 1: Figure 1D). However, we observed no difference in total endogenous DYRK1A catalytic activity using high-performance liquid chromatography (HPLC) [4] (Additional file 1: Figure 1E). We performed western blots using the D1694 antibody targeting the N-terminal region of DYRK1A (named after α-DYRK1A-Nter) and we observed decreased levels of the full-length form of DYRK1A (DYRK1A_*FL*_, 90 kDa) associated to an increase of the truncated form (DYRK1A_*T*_, 50 kDa) in the hippocampus of AD patients (Additional file 1: Figure 1F). We then further characterized expression of the different forms of DYRK1A by immunohistochemistry on hippocampal slices. Combining α-DYRK1A-Cter and α-DYRK1A-Nter antibodies (detailed in Additional file 2: Figure 2) we evidenced decreased DYRK1A staining intensity using both antibodies in AD cases compared to controls (Additional file 1: Figure 1G,H). In addition, an astrocytic staining by the α-DYRK1A-Nter antibody was observed, as confirmed by double-immunofluorescence and confocal laser (Additional file 1: Figure 1I). Altogether, these results indicate that DYRK1A undergoes a proteolytic processing in human AD hippocampus leading to the decrease of DYRK1A_*FL*_ and the accumulation of DYRK1A_*T*_ thus confirming previous study. Particularly, we here identified that this pathological mechanism is located at least in part in astrocytes cells and that it does not affect the level of DYRK1A kinase activity.

### Leucettine L41 prevents in vitro DYRK1A proteolysis and limits its interaction with STAT3α

**Fig. 1:**
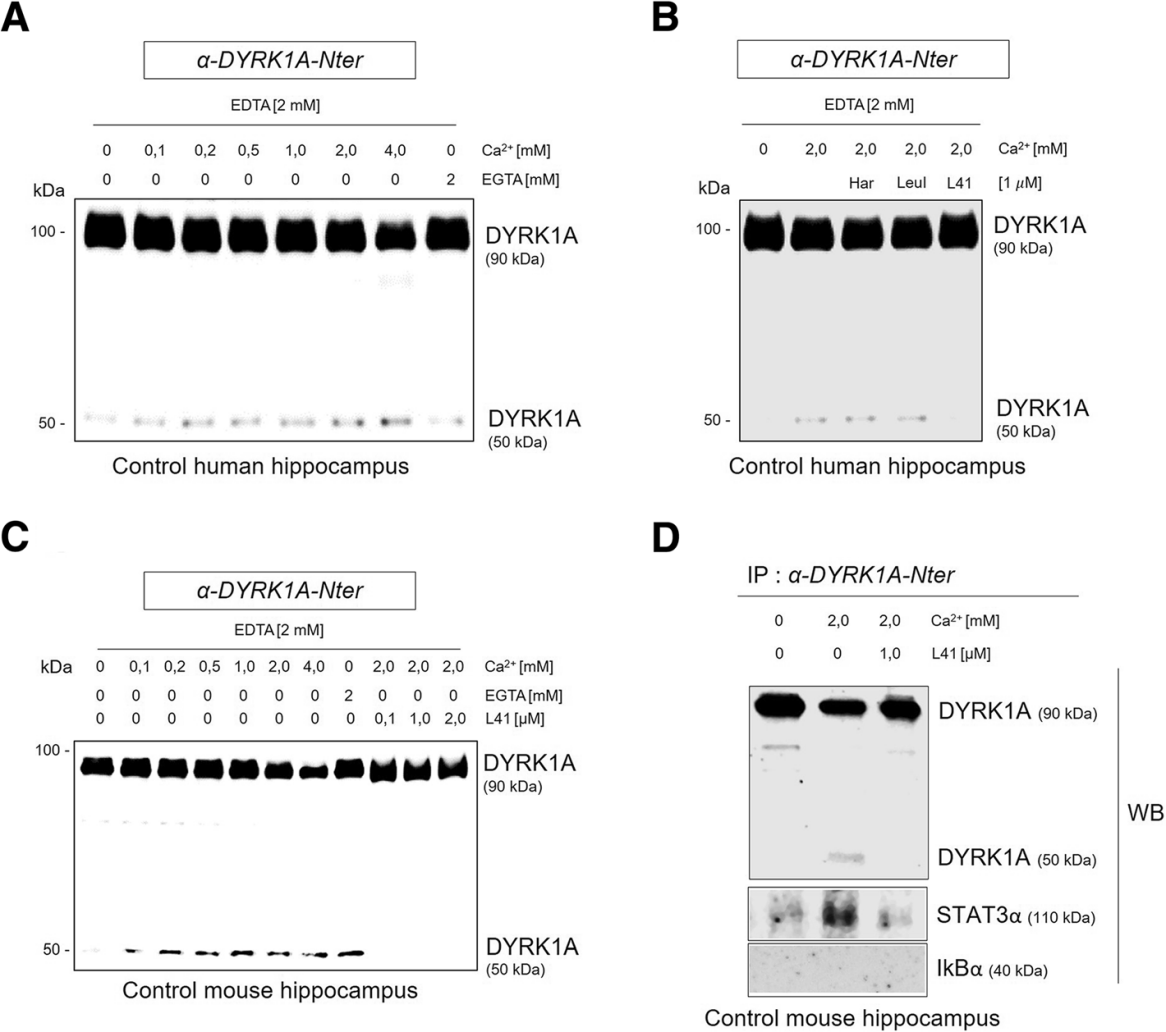
Identification of Leucettine L41 as a DYRK1A proteolysis inhibitor*.* **a** Control human hippocampus extract was incubated at 30 °C during 10 min with various concentrations of CaCl_2_ (0 to 4,0 mM) or with 2 mM of EGTA. Proteins were then analyzed by western blot using the *α-DYRK1A-Nter* antibody. **b** Control human hippocampus extract was incubated with 0 mM of CaCl_2_ or 2 mM of CaCl_2_ and various pharmacological compounds including Harmine (har), Leucettine LeuI (LeuI) or Leucettine L41 (L41). Proteins were analyzed by western blot using the *α-DYRK1A-Nter* antibody. **c** Control mouse (C57Bl6) hippocampus extract was incubated at 30 °C during 10 min with various concentrations of CaCl_2_ (0 to 4,0 mM) or/with 2 mM of EGTA or/with 2 mM of CaCl_2_ and various concentrations of Leucettine L41 (L41) (0,1 to 2 *μ*M). Proteins were analyzed by western blot using the *α-DYRK1A-Nter* antibody. **d** Control mouse hippocampus extract was incubated at 30 °C during 10 min with 0 mM of CaCl_2_ or 2 mM of CaCl_2_ or 2 mM of CaCl_2_ with L41 at 1 *μ*M. Protein extracts were then immunoprecipitated with the *α-DYRK1A-Nter* antibody overnight at 4 °C and immunoprecipitated protein extract were analyzed by western blot using *α-DYRK1A-Nter*, STAT3α and IkBα antibodies

We then tested known DYRK1A inhibitors including Harmine [13], Leucettine LeuI and Leucettine L41 (also named L41) for their ability to alter DYRK1A proteolysis. In vitro assays were performed as previously described [23]. Human hippocampus extracts were incubated with different concentrations of calcium. A Ca^2+^ dose-dependent decrease of DYRK1A_*FL*_ and increase DYRK1A_*T*_ were observed while the Ca^2+^ chelating agent EGTA was used as a control (Fig. 1a). Only L41 (1 μM) efficiently inhibited DYRK1A proteolysis, maintaining DYRK1A_*FL*_ levels and preventing DYRK1A_*T*_ formation (Fig. 1b). Similar results were obtained using mouse hippocampus extracts (Fig. 1c) suggesting that Leucettine L41 is able to prevent in vitro the DYRK1A proteolysis in both human and mouse proteins extracts.

In AD hippocampus, DYRK1A proteolysis does not modify its global kinase activity (see Additional file 1: Figure 1E). To assess whether DYRK1A C-terminal fraction is necessary for its selectivity, we performed DYRK1A immunoprecipitation using the α-DYRK1A-Nter antibody on mouse extracts incubated or not with Ca^2+^ (2 mM) and L41. We then performed western-blots using STAT3α and IkBα antibodies (Fig. 1d). No binding between both DYRK1A forms and IkBα was revealed. In contrast, we observed a modest interaction between DYRK1A_*FL*_ and STAT3α in absence of Ca^2+^. This interaction increased in presence of Ca^2+^ and was altered by the addition of L41 (Fig. 1d). These compelling data suggest that DYRK1A_*T*_ has a higher affinity toward STAT3α compared to DYRK1A_*FL*_.

### Leucettine L41 treatment prevents proteolytic processing of DYRK1A in APP/PS1 mice

**Fig. 2:**
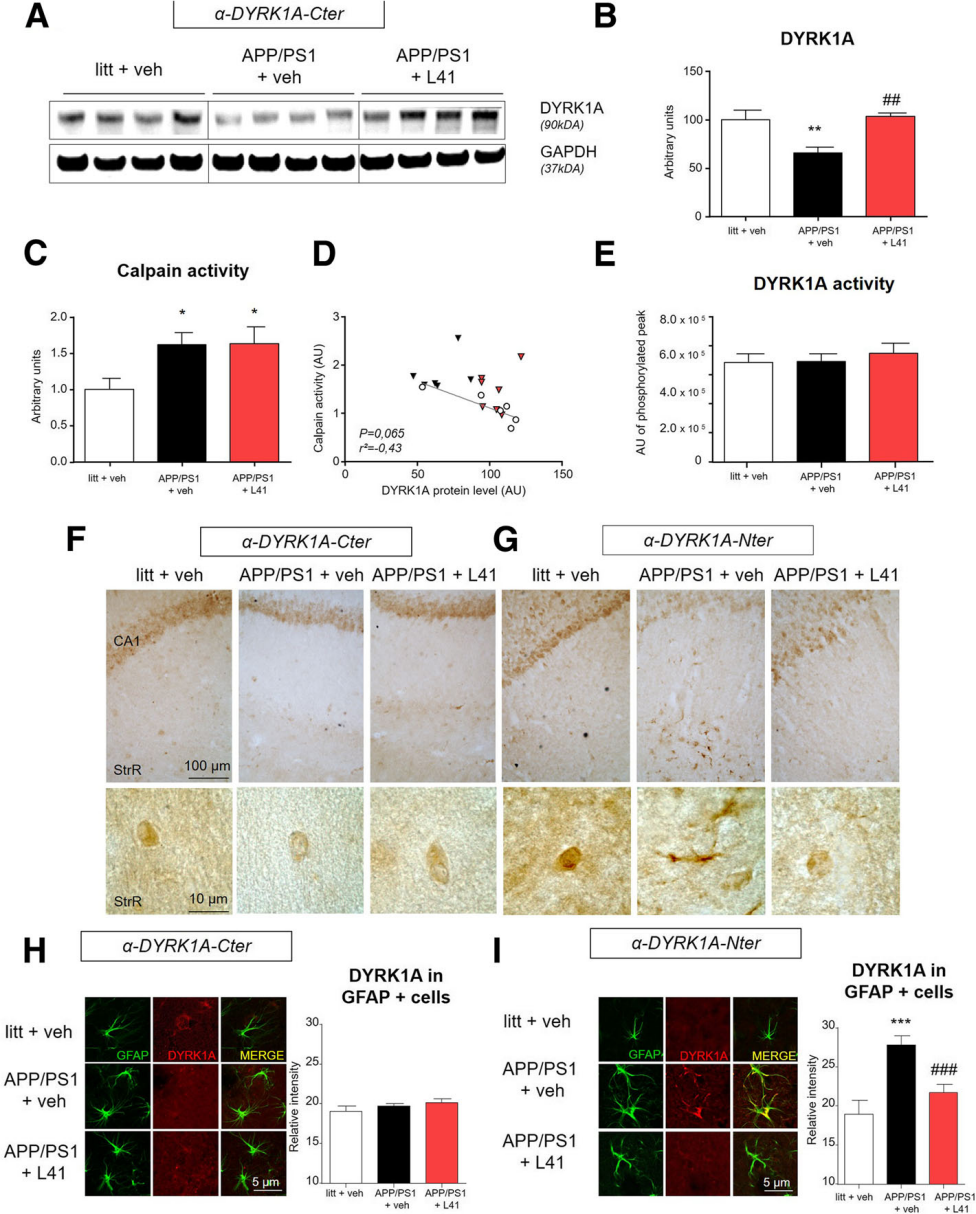
L41 treatment prevents DYRK1A proteolysis in APP/PS1 mice hippocampus. **a**, **b** Western blot of hippocampus from APP/PS1 mice or littermates treated with vehicle or L41, showing lower levels of DYRK1A (90 kDa) immunoblotting with the *α-DYRK1A-Cter* antibody in vehicle-treated APP/PS1 mice (*n* = 6) compared to littermates (n = 6) (One-way ANOVA, *p* < 0.005). DYRK1A protein levels in L41-treated APP/PS1 mice (*n* = 7) were higher than in vehicle-treated APP/PS1 mice (One-way ANOVA, *p* < 0.005) and similar to what observed in littermates (One-way ANOVA, *ns*). **c** Calpain activity assessed by a fluorescent method, showing higher calpain activity in hippocampus from both vehicle-treated (n = 6) and L41-treated APP/PS1 mice (n = 7) compared to littermates (n = 6) (One-way ANOVA, *p* < 0.05 for both). There was no significant difference between L41-treated and vehicle-treated APP/PS1 mice (One-way ANOVA, *ns*). **d** DYRK1A protein levels did not correlate with calpain activity (r^2^ = 0.43; *ns*). **e** HPLC assay for total endogenous DYRK1A activity showing no differences between hippocampus from littermates (*n* = 9) and vehicle- or L41-treated APP/PS1 mice (*n* = 9 and 8, respectively) (One-way ANOVA, *ns*). **f** Representative images from immunohistochemical staining using the *α-DYRK1A-Cter* antibody, of hippocampal slices from littermates, vehicle- and L41-treated APP/PS1 mice, showing neuronal staining in the CA1 and Stratum Radiatum (StrR) regions (see enlargement at the bottom). **g** Representative images from immunohistochemical staining, using the *α-DYRK1A-Nter* antibody, of hippocampal slices, showing neuronal staining for L41-treated APP/PS1 mice and littermates in the CA1 and Stratum Radiatum (StrR) regions. Neuronal staining was observed in both the CA1 and StrR regions, whereas additional astrocyte staining was mostly observed in the Stratum Radiatum (StrR) region of vehicle-treated APP/PS1 mice. **h** Laser confocal microscopy showing double staining using *α-DYRK1A-Cter* (red) and anti-GFAP (green) antibodies. There were no differences in *α-DYRK1A-Cter* staining in GFAP positive cells between littermates (n = 6, astrocytes = 73), vehicle- (n = 6, astrocytes = 90), and L41-treated APP/PS1 mice (n = 6, astrocytes = 85) (One-way ANOVA, *ns*). **i** Laser confocal microscopy showing double staining using *α-DYRK1A-Nter* antibody (red) and anti-GFAP (green). *α-DYRK1A-Nter* staining was higher in the GFAP positive area in vehicle-treated APP/PS1 mice (n = 6, astrocytes = 105) compared to littermates (n = 6, astrocytes = 50) (One-way ANOVA, *p* < 0.0005). *α-DYRK1A-Nter* immunoreactivity was lower in the GFAP positive area of L41-treated (n = 6, astrocytes = 83) than vehicle-treated APP/PS1 mice (One-way ANOVA, *p* < 0.0005). Data represent the mean ± SEM and were analyzed by one-way ANOVA followed by Tukey’s post hoc test. Significant differences between littermates and vehicle-treated APP/PS1 mice are indicated by *p < 0.05, **p < 0.005 and ***p < 0.0005. Significant differences between vehicle- and L41-treated APP/PS1 mice are indicated by ^#^*p* < 0.05, ^##^*p* < 0.005 and ^###^*p* < 0.0005

We then aimed to determine whether L41 could modify DYRK1A proteolysis in vivo with potential consequences on AD phenotype. We used 13-month-old APP/PS1 mice with severe and established AD pathology. These mice present a positive correlation between Aβ accumulation and calpain activity [43]. The treated group received intraperitoneal injections of L41, 5 days a week during one month. Littermates and another cohort of APP/PS1 mice received injections of the vehicle solution. Vehicle-treated APP/PS1 mice had lower DYRK1A_*FL*_ levels than wild type littermates by western blot analysis using α-DYRK1A-Cter (*p* < 0.005) (Fig. 2a, b). They showed a corresponding two-fold increase in calpain activity (*p* < 0.005) (Fig. 2c), leading to a negative Spearman correlation with DYRK1A_*FL*_ protein levels (correlation coefficient r = − 0.65, *p* < 0.021) (Additional file 3: Figure 3A). L41 treatment fully restored DYRK1A_*FL*_ protein levels in APP/PS1 mice to wild-type levels (*p* < 0.005 vs vehicle-treated APP/PS1) (Fig. 2a, b), independently of a change in calpain activity (*p* < 0.005 vs vehicle-treated littermates) (Fig. 2c). Thus, there was no significant correlation between DYRK1A_*FL*_ protein levels and calpain activity (correlation coefficient − 0.43, ns) (Fig. 2d). In contrast, protein levels of various kinases including GSK3β exhibited no change between littermates, vehicle-treated APP/PS1 and L41-treated APP/PS1 (Additional file 3: Figure 3B). No differences in total DYRK1A kinase activity was observed among the three experimental groups (Fig. 2e). Levels of phosphorylated forms of Tau protein at Thr 212 or Thr 231 and APP protein at Thr 668 which are described as epitopes targeted by DYRK1A were not decreased by Leucettine L41 treatment (Additional file 4: Figure 4A and B).

Immunohistochemical analysis using both antibodies (α-DYRK1A-Cter and α-DYRK1A-Nter) showed lower DYRK1A staining in the hippocampi of vehicle-treated APP/PS1 mice compared to littermates for both antibodies, confirming biochemical analysis. Strikingly, treatment of APP/PS1 mice with L41 restored DYRK1A staining levels in the hippocampus to those of wild-type mice. Most pyramidal neurons in the CA1 region and interneurons in the Stratum Radiatum (StrR) exhibited DYRK1A staining in littermates and APP/PS1 mice treated or not with L41 (Fig. 2f and Fig. 2g, respectively). In contrast, additional staining by the α-DYRK1A-Nter antibody was observed in the cytosol of hippocampal astrocytes of vehicle-treated APP/PS1 mice (Fig. 2g). This was confirmed by double-immunofluorescence and confocal microscopy using both anti-DYRK1A antibodies and an anti-GFAP antibody (Fig. 2h and i). The α-DYRK1A-Cter antibody, which targets only the DYRK1A_*FL*_ forms, showed only marginal co-localization between GFAP and DYRK1A_*FL*_ in all mice groups, as revealed by the level of DYRK1A_*FL*_ in GFAP-positive cells, which was the same for all three groups (Fig. 2h). The α-DYRK1A-Nter antibody, which targets both DYRK1A_*FL*_ and DYRK1A_*T*_, showed strong co-localization between GFAP and DYRK1A_*FL*_/DYRK1A_*T*_ in the hippocampi of vehicle-treated APP/PS1 mice. In contrast, there was only negligible co-localization in wild-type littermates and Leucettine L41-treated APP/PS1 mice. The level of DYRK1A in GFAP-positive cells of vehicle-treated APP/PS1 mice was higher than that in GFAP-positive cells of littermates and L41-treated APP/PS1 mice (*p* < 0.0005 for both) (Fig. 2i). These findings confirm our previous results in human samples and indicate that L41 can prevent in vivo DYRK1A processing without changing DYRK1A or calpain activities.

### Leucettine L41 treatment prevents STAT3α phosphorylation and reduces pro-inflammatory cytokines release in APP/PS1 mice

**Fig. 3:**
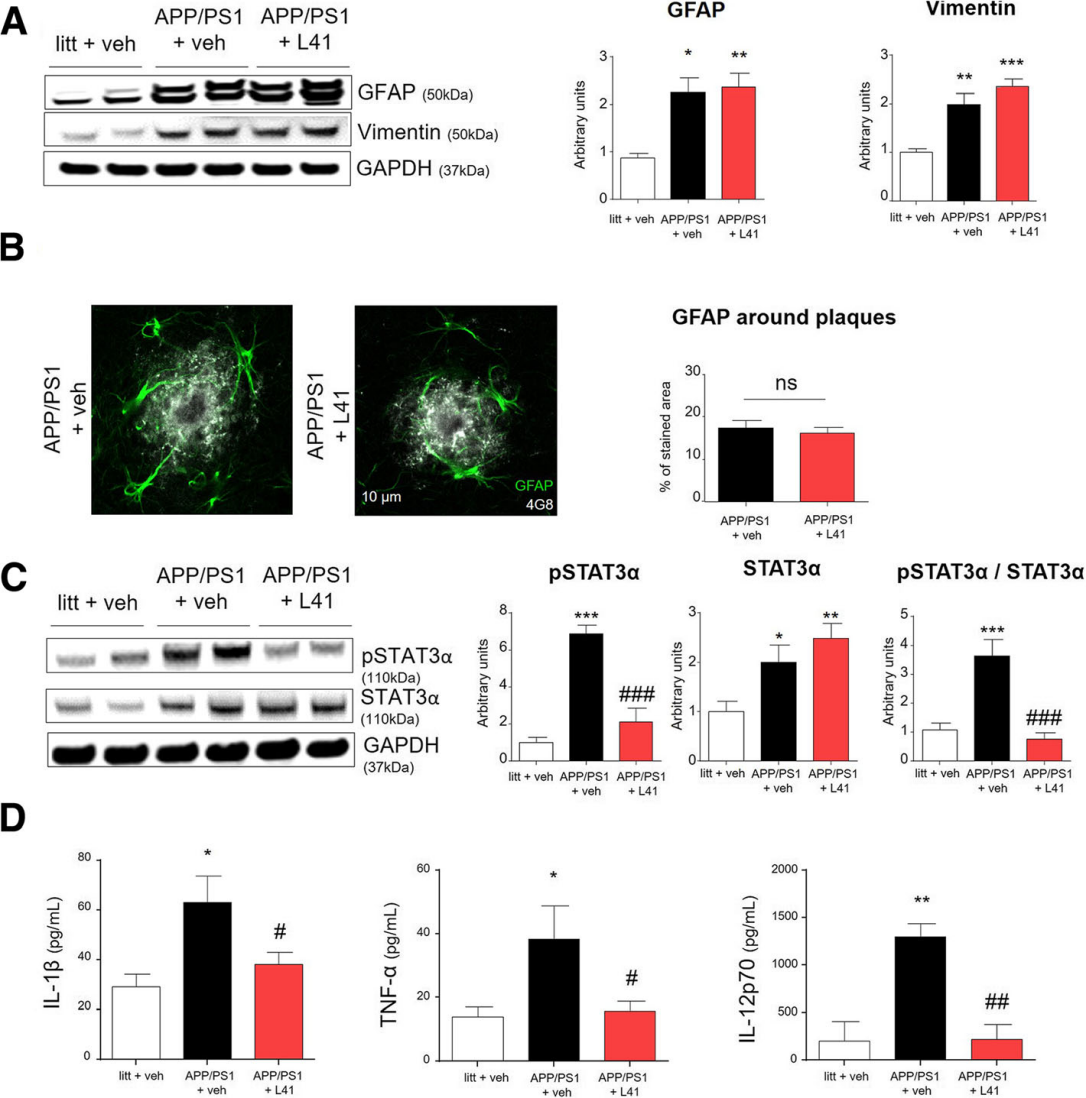
L41 treatment prevents DYRK1A_*T*_ accumulation in astrocytes and decreases phosphorylation of STAT3*α* and the release of pro-inflammatory cytokines. **a** Representative western blot of mouse hippocampus showing higher levels of GFAP and vimentin in vehicle- (*n* = 6) and L41- (*n* = 7) treated APP/PS1 mice than in littermates (*n* = 6) (One-way ANOVA; GFAP: *p* < 0.05 and *p* < 0.005, respectively; vimentin: *p* < 0.005 and *p* < 0.0005, respectively). There were no statistical differences between L41- and vehicle-treated APP/PS1 mice (One-way ANOVA, *ns* and *ns*). **b** Laser confocal microscopy showing double staining, using anti-4G8 (white) and anti-GFAP (green) antibodies, of hippocampal slices from vehicle- (*n* = 6, plaques = 39) or L41- (*n* = 6, plaques = 38) treated APP/PS1 mice. There were no differences in GFAP (green) immunoreactivity around amyloid plaques (white) between vehicle- and L41-treated APP/PS1 mice (*t*-test, *ns*). **c** Representative western blot of hippocampus from mice showing higher phospho-STAT3*α* / STAT3*α* ratio in vehicle- treated APP/PS1 mice compared to littermates and L41-treated APP/PS1 mice (One-way ANOVA; pSTAT3*α*: *p* < 0.0005 and *p* < 0.0005, respectively; STAT3*α*: *p* < 0.05 and ns, respectively; pSTAT3*α* / STAT3*α*: *p* < 0.0005 and *p* < 0.0005, respectively) (**d**) ELISA quantification (MSD immunoassay) in samples from hippocampus of littermates, vehicle- and L41-treated APP/PS1 mice (*n* = 4, 6, and 7 mice per group, respectively). Vehicle-treated APP/PS1 mice had higher IL-1*β*, IL-12 (IL-12p70) and TNF-*α* concentrations than in littermates (One-way ANOVA: IL-1*β* and TNF-*α*, *p* < 0.05; IL-12p70, *p* < 0.005). L41-treated APP/PS1 mice had lower IL-1*β*, IL-12 and TNF-*α* concentrations than vehicle-treated APP/PS1 mice (One-way ANOVA: IL-1*β*, and TNF-*α*, *p* < 0.05; IL-12p70, *p* < 0.005). Data represent the mean ± SEM and were analyzed by one-way ANOVA followed by Tukey’s post hoc test or Student’s *t*-test. Significant differences between littermates and vehicle-treated APP/PS1 mice are indicated by *p < 0.05, **p < 0.005 and ***p < 0.0005. Significant differences between vehicle- and L41-treated APP/PS1 mice are indicated by ^#^*p* < 0.05, ^##^*p* < 0.005 and ^###^*p* < 0.0005

After showing in vitro an increased affinity of DYRK1A_*T*_ toward STAT3ɑ (see Fig. 1), we evaluated L41 influence on astrocytes and STAT3ɑ phosphorylation state in APP/PS1 mice. We first assessed GFAP and vimentin protein levels in the hippocampus by western blot. As expected, both GFAP and vimentin levels were increased in APP/PS1 mice hippocampi but were not affected by L41 treatment (*p* < 0.05 and *p* < 0.005 respectively) (Fig. 3a). We confirmed no alteration of the astrocytes recruitment around the amyloid plaques in APP/PS1 treated with L41 (Fig. 3b). STAT3ɑ as its phosphorylated form [phospho- STAT3α (Tyr705)] was increased in vehicle-treated APP/PS1 relative to littermates mice (*p* < 0.05 and *p* < 0.0005 respectively). Interestingly, L41-treated APP/PS1 exhibited lower phospho-STAT3α levels compared to vehicle-treated APP/PS1 mice (*p* < 0.0005), thereby leading to a restored ratio pSTAT3ɑ/STAT3ɑ similar to littermates (Fig. 3c). STAT3 is an important signaling molecule for cytokines and growth factor receptors production [7, 18] and has been associated with pro-inflammatory cytokines expression such as IL-1β and TNF-ɑ [6]. We measured by ELISA these pro-inflammatory cytokines levels released in part by reactive astrocytes. Higher concentrations of IL-1β, IL-12 and TNF-ɑ were measured in APP/PS1 mice compared to littermates (*p* < 0.05, *p* < 0.005, and *p* < 0.05, respectively). These levels were reversed by L41 treatment (*p* < 0.05, *p* < 0.005, and *p* < 0.05, respectively, for L41 vs vehicle-treated APP/PS1 mice) (Fig. 3d). Collectively, these data demonstrate that DYRK1A_*T*_ forms participate to astrocyte inflammatory cytokines production through a STAT3ɑ pathway activation.

### Leucettine L41 treatment promotes microglia recruitment around amyloid plaques in APP/PS1 mice

**Fig. 4:**
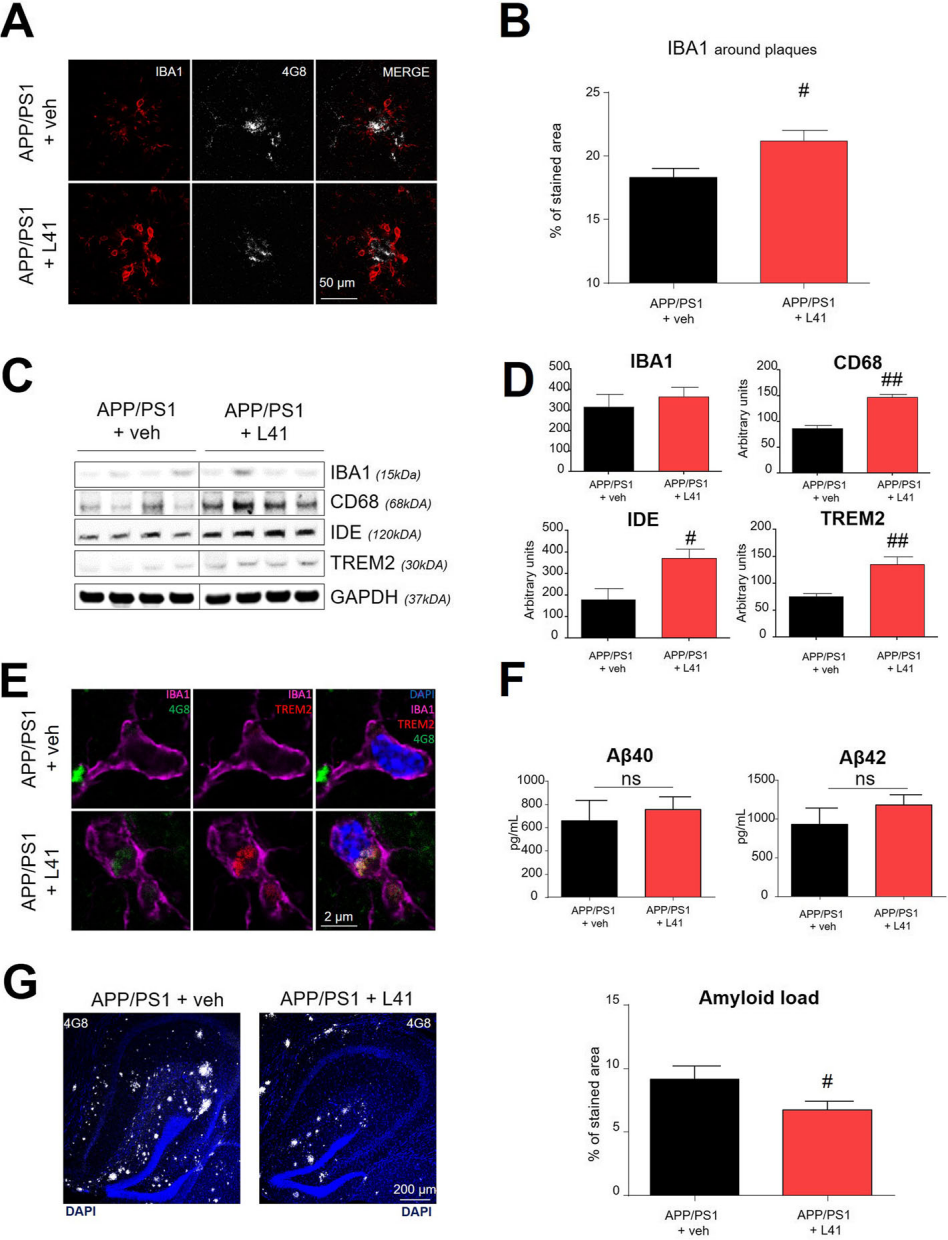
L41 treatment promotes microglial cells recruitment leading to a decrease in aggregated amyloid load. **a** Laser confocal microscopy showing double staining, using 4G8 antibody (white) and anti-IBA1 (red), of hippocampal slices from vehicle- (*n* = 6, plaques = 79) or L41- (*n* = 6, plaques = 77) treated APP/PS1 mice. **b** Increased IBA1 immunoreactivity around amyloid plaque of L41-treated APP/PS1 mice, compared to vehicle-treated APP/PS1 mice, was observed (*t*-test, *p* < 0.05). **c** Western blot of hippocampus from vehicle (*n* = 6) or L41 (*n* = 7) treated APP/PS1 mice, showing levels of IBA1, CD68, IDE, and TREM2. **d** IBA1 protein levels were not statistically different in L41-and vehicle-treated APP/PS1 mice (*t*-test, *ns*). CD68, IDE, and TREM2 protein levels were increased in L41-treated compared to vehicle-treated APP/PS1 mice (CD68: *t*-test, *p* < 0.005; IDE: *t*-test, *p* < 0.05; TREM2: *t*-test, *p* < 0.005). **e** Laser confocal microscopy showing IBA1 positive cells with increased TREM2 protein and colocalization with A*β* species (4G8) in L41-treated compared to vehicle-treated APP/PS1 mice. **f** ELISA quantification of soluble A*β*_40_ and A*β*_42_ (MSD immunoassay) in vehicle- and L41-treated APP/PS1 mice hippocampus (*n* = 6/7 mice per group). There was no statistical differences between L41- and vehicle-treated APP/PS1 mice. **g** Laser confocal microscopy showing double staining with 4G8 antibody (white) and DAPI (blue), of hippocampal slices from vehicle- (*n* = 6) or L41- (*n* = 6) treated APP/PS1 mice. Area covered by the plaques was decreased in the hippocampus of L41-treated compared to vehicle-treated APP/PS1 mice (*t*-test, *p* < 0.05). Data represent the mean ± SEM and were analyzed by one-way ANOVA, followed by Tukey’s post hoc test or Student’s *t*-test. Significant differences between vehicle- and L41-treated APP/PS1 mice are indicated by ^#^*p* < 0.05, ^##^*p* < 0.005, and ^###^ *p* < 0.0005

In response to brain injury, crosstalk between astrocytes and microglial cells is driven in part by inflammatory mediators [28]. Using confocal microscopy and double-immunofluorescence, we observed increased activated microglia (IBA1 staining) around amyloid-β plaques in L41-treated APP/PS1 mice compared to vehicle-treated APP/PS1 mice (*p* < 0.05) (Fig. 4a-b). Western blot analysis did not reveal differences in IBA1 protein levels. However, levels of CD68, IDE and TREM2, microglial markers involved in amyloid-β clearance, were increased in L41-treated APP/PS1 mice (*p* < 0.0005, *p* < 0.05, and *p* < 0.05, respectively) (Fig. 4c-d). TREM2 increase was confirmed in microglial cells of L41-treated APP/PS1 mice by immunohistochemistry and was associated with an amyloid staining suggesting that L41 promotes amyloid-β clearance by microglia (Fig. 4e). Because of the well-known roles of CD68, IDE and TREM2 in Aβ phagocytosis and degradation [19, 38], we evaluated the effects of L41 on soluble and aggregated forms of Aβ in APP/PS1 mice hippocampi. Neither soluble Aβ_40_ and Aβ_42_ concentrations were significantly different between L41- and vehicle-treated APP/PS1 mice (Fig. 4f). In contrast, we observed a significant decrease of the amyloid-β load in the hippocampi of L41-treated APP/PS1 mice (*p* = 0.049) (Fig. 4g). Altogether, these results demonstrate that L41 treatment promotes microglia recruitment around Aβ plaques, increases levels of proteins involved in amyloid-β clearance and leads to decreased amyloid load.

### Leucettine L41 treatment rescues synaptic and memory impairments in APP/PS1 mice

**Fig. 5:**
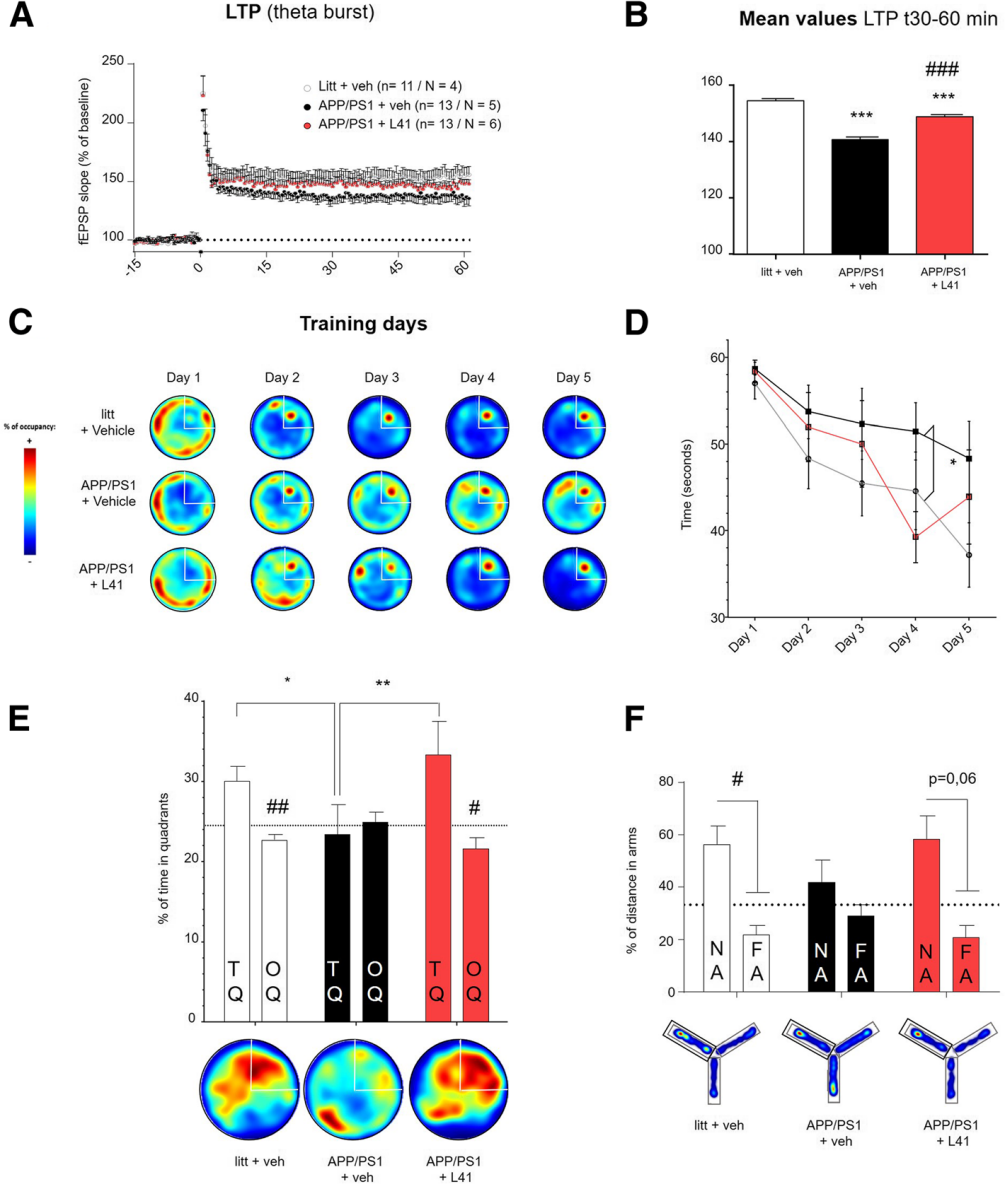
L41 treatment improves synaptic plasticity and prevents behavioral deficits in APP/PS1 mice. **a**, **b** Long-term potentiation (LTP) was induced by delivering theta-burst stimulations to hippocampal CA3-CA1 synapses after 20 min of baseline recordings. Slices from vehicle-treated APP/PS1 mice exhibited deficits in LTP expression relative to littermates. The magnitude of LTP observed in L41-treated APP/PS1 mice was higher than that in vehicle-treated APP/PS1 mice, but lower than in littermates. **b** Summary bar graph showing the average potentiation represented as the percentage from 30 to 60 min after theta-burst stimulation. Slices from vehicle-treated APP/PS1 mice showed a mean deficit of LTP (137.7 ± 0.163) relative to that of littermates (153.5 ± 0.211) (One-way ANOVA, *p* < 0.0005). The mean LTP of L41-treated APP/PS1 mouse slices (146.8 ± 0.243) was higher than that of vehicle-treated APP/PS1 mice (One-way ANOVA, *p* < 0.0005), but lower than that of littermates (One-way ANOVA, *p* < 0.0005). *n* = number of slices, N = number of mice. Significant differences between littermates and APP/PS1 injected with vehicle are indicated by *** *p* < 0.0005. Significant differences between vehicle- and L41-treated APP/PS1 mice are indicated by ^###^*p* < 0. (**c**, **d**, **e**) Spatial learning and long-term memory were evaluated using the Morris water maze paradigm on littermates (*n* = 12), vehicle- (n = 7) and L41- (n = 7) treated APP/PS1 mice. **c** Representative occupancy plots during acquisition show a more random search strategy for vehicle- than L41-treateted APP/PS1 mice and littermates. **d** Escape latency of vehicle-treated littermate controls or vehicle- or L41-treated APP/PS1 mice. The time to reach the platform was different between the groups (Two-way ANOVA: Group effect: F2.110 = 3.68, *p* = 0.028; Time effect: F4.110 = 7.23, *p* < 0.0001; Group x Time interaction: F8.110 < 1, ns). Vehicle-treated APP/PS1 mice were impaired relative to vehicle-treated littermate controls (*p* = 0.02). L41-treated APP/PS1 mice were statistically indistinguishable from littermate controls (*p* = *ns*). Significant differences between littermates and vehicle-treated APP/PS1 mice are indicated by ^*^p < 0.05. **e** Probe trial performance at 72 h. (Two-way ANOVA, Group effect: F2.46 = 1.315, p = ns; quadrant effect: F1.46 = 12.58, *p* = 0.009; Group x quadrant interaction effect: F2.46 = 5.27, *p* = 0.0087). Vehicle-treated APP/PS1 mice were impaired relative to vehicle-treated littermates (*p* < 0.05). L41 treatment rescued this memory impairment (*p* < 0.005), confirmed by a preference for the trained target quadrant. Target quadrant (TQ) and other quadrants (OQ). Bottom: representative occupancy plots during 72 h-probe test show a random search strategy for vehicle-treated APP/PS1 mice, in contrast to both vehicle-treated littermates and L41-treated APP/PS1 mice. Significant differences between the groups are indicated by *p < 0.05 and ** *p* < 0.005. Significant differences between the quadrants are indicated by ^#^*p* < 0.05 and ^##^ *p* < 0.005. Data represent the mean ± SEM and were analyzed by one-way or two-way ANOVA followed by Tukey’s post hoc test. **f** Working memory was evaluated using the Y-maze paradigm in littermates (*n* = 9) and vehicle- (*n* = 8), or L41- (*n* = 5) treated APP/PS1 mice. The distance covered by the mice in the arms during the evaluation phase was different between each arm (Two-way ANOVA: Group effect: F2.19 = 1.232; *ns*; Arm effect: F1.19 = 15.31, *p* < 0.005; Group x Arm interaction: F2.19 = 1 .232, *ns*). Vehicle-treated APP/PS1 mice did not cover significantly more distance in the NA (new arm) than the OA (other arms, including starting and familiar arms) (Two-way ANVOVA, *ns*). Littermates and L41-treated APP/PS1 mice covered more distance in the new arm than in the other arms (*p* < 0.05 for both). Significant differences between distance covered in the arms are indicated by ^#^ *p* < 0.05

APP/PS1 mice exhibit decreased long-term potentiation (LTP) of synaptic transmission associated with impaired learning and memory [12]. It has been reported that neuroinflammation mechanisms and in particular immune diffusible mediators such as cytokine influence synaptic transmission, plasticity and thereby memory [2, 10]. We explored whether L41 treatment (daily injection from age 13 to 14 months) could rescue LTP and cognitive deficits. As expected, APP/PS1 treated with vehicle (*n* = 13 slices/*N* = 5 mice) exhibited impaired LTP relative to littermates treated with the vehicle solution (*n* = 11 slices/*N* = 4 mice) (*p* < 0.0005) (Fig. 5a, b). L41-treated APP/PS1 mice exhibited improved LTP (*p* < 0.0005) relative to APP/PS1 mice that received vehicle injections. LTP generated in L41-treated APP/PS1 mice was still significantly lower compared to littermates (*p* < 0.0005), suggesting that synaptic plasticity measured by the LTP was partially rescued by the L41 treatment. To evaluate whether improved synaptic plasticity was also reflected at the behavioral level, we tested the mice by the Morris water-maze and the Y-maze tasks (Fig. 5c-f). Spatial learning and long-term memory were assessed using the Morris water maze paradigm (Fig. 5c-e). All mice progressively learned the platform position during the learning sessions, as demonstrated by a decrease in the time required to reach the platform over the five days of training. Vehicle-treated APP/PS1 mice exhibited a reduced learning ability relative to littermates (*p* = 0.02), whereas L41-treated APP/PS1 mice were statistically undistinguishable from littermate controls (ns) (Fig. 5c, d). During the 72-h probe test, vehicle-treated APP/PS1 mice spent less time in the target quadrant than littermates (*p* < 0.05) (Fig. 5e). In contrast, L41-treated APP/PS1 mice spent more time in the target quadrant compared to vehicle-treated APP/PS1 mice (*p* < 0.005) but not different compared to littermates (ns). We also used the Y-maze paradigm to evaluate the spatial working memory which is mediated by hippocampus but also prefrontal cortex [47](Fig. 5f). Vehicle-treated APP/PS1 mice did not travel more distance in the new arm compared to the familiar starting arm, suggesting an impaired working memory (ns). In contrast, vehicle-treated littermates exhibited a spatial preference for the new arm (*p* < 0.05) whereas L41-treated APP/PS1 mice tended to exhibit similar behavior (*p* = 0.06) (Fig. 5f). Taken together, these results suggest that L41 treatment alleviates synaptic plasticity impairments and rescues memories defects in aged APP/PS1 mice (14-month-old) with well-established cognitive deficits.

## Discussion

The present study provides compelling evidence for DYRK1A involvement in AD and describes a new mechanism through which DYRK1A modulation contributes to AD pathology. We first describe a proteolytic processing of DYRK1A in the hippocampus of AD patients and APP/PS1 mice reducing level of full-length form of DYRK1A (DYRK1A_*FL*_) and producing truncated forms (DYRK1A_*T*_). The increase of DYRK1A kinase activity was suspected to contribute to AD. However, we demonstrate that this proteolysis is not associated with modification of the global DYRK1A kinase activity but affect its specificity. DYRK1A_*T*_ forms exhibit increased affinity toward STAT3α, an activator of neuroinflammation. We then show that inhibiting DYRK1A proteolysis through the peripheral administration of Leucettine L41 in APP/PS1 mice is associated with increased number of phagocytic-microglia around amyloid-β deposits and reduction of the plaque load. This is associated to improved synaptic plasticity and reduced cognitive and memory deficits in APP/PS1 mice. Specific inhibitors of DYRK1A proteolysis could be therapeutic interest for AD.

The *DYRK1A* gene is located on chromosome 21 (21q22.2), a region known as the Down- Syndrome Critical Region (DSCR) [9]. People living with Down Syndrome (DS) have higher prevalence to develop AD pathology primarily due to overexpression of the *APP* gene on chromosome 21 [17]. In addition, various evidence supports DYRK1A as a potential key player in AD progression and as a valid therapeutic target for AD [3, 35]. However, no direct link has been shown between the kinase activity of DYRK1A and AD. Recently, decreased DYRK1A_*FL*_ and increased DYRK1A_*T*_ forms have been reported in the frontal and temporal cortex of AD patients (Braak V-VI/Tangles score = 14) through upregulated calpain I activity, the major calpain isoform in brain [23]. We confirm this observation in hippocampus from severely affected AD patients (Braak V-VI, Thal IV-V). We show for the first-time decreased levels of DYRK1A_*FL*_ forms in APP/PS1 mice, together with increased DYRK1A_*T*_ forms and increased calpain activity.

DYRK1A has a long kinase domain, followed by a PEST region, and histidine-repeat and serine/threonine-rich domains [24]. PEST sequences are rich in the amino acids proline (P), glutamate (E), serine (S) and threonine (T), and their presence is correlated with rapid protein turnover due to proteasome-mediated destruction [32]. Proteolysis of DYRK1A by calpains probably occurs within the PEST domain [23] as shown in other proteins [32]. Beyond the PEST sequence, the C-terminus domain is the target of other proteins which negatively modulate DYRK1A kinase activity [49]. In numerous protein kinases, non-catalytic domains participate in the kinase specificity [44]. In our study, we provide evidences that the deletion of the C-terminal region does not affect kinase activity of DYRK1A but increases its affinity toward STAT3α.

Several molecules able to reduce DYRK1A kinase activity have been developed [31]. One of them, Leucettine L41 (L41) is a synthetic analogue of the marine sponge alkaloid Leucettamine B, identified as an inhibitor of DYRKs/CLKs [8, 41]. Although L41 prevents DYRK1A proteolysis in APP/PS1 mice hippocampus, we showed that this compound does not alter DYRK1A (and Calpain) activity. Thus, several hypotheses can be considered to explain L41 effect on DYRK1A: (i) L41 could prevent the interaction between DYRK1A and calpains by inducing a conformational change in the kinase, (ii) the DYRK1A/L41 complex could change its intracellular location and thereby be isolated from calpains, or (iii) the catalytic activity of DYRK1A could be required for calpain-mediated cleavage. Further experiments are still needed to better understand the action of L41 on DYRK1A regulation.

Consequently to the prevention of DYRK1A, we showed a decrease of STAT3α phosphorylation (pSTAT3α) in APP/PS1 mice. STAT3ɑ is a transcription factor and a major regulator of cytokine production [18]. The tyrosine phosphorylation is required for its activation and STAT3α are remarkably activated in APP/PS1 mice [7]. Inhibition of STAT3α phosphorylation attenuates Aβ-induced neuronal death [45]. Our results indicate that a normalization of pSTAT3ɑ levels by L41 restores pSTAT3α/STAT3α ratio and may participate to the following events: (i) decreased release of key inflammatory mediators such as IL-1β, TNF-*α* and IL-12, (ii) increased microglial cells recruitment around amyloid plaques, and (iii) decrease of the amyloid-β burden. The immune system, driven by inflammatory mediators, influences AD progression [19]. In particular, TNF-α has been described to have a major impact in AD. Indeed, increased TNF-α in serum is associated with a worsen cognitive decline in AD [21] and elevated concentrations of TNF-α in CSF increase the probability to evolve from a mild cognitive impairment (MCI) stage to dementia [42]. In addition, these molecules have a strong impact on microglial dysfunction [5, 40]. Emerging evidences suggest that microglial activation plays a crucial role in AD [16]. Activated microglia have receptors that can uptake and clear Aβ and this may limit the formation of plaques through phagocytosis of Aβ species [38]. In APP/PS1 mice, proinflammatory cytokines (IL-1β and TNF-α) increase in concentration with age and down regulate genes involved in amyloid-β clearance [20]. Therefore, microglia become progressively dysfunctional and display altered activation as the disease progresses. Our data show that L41 treatment in APP/PS1 mice promotes a moderate reduction of the amyloid load, which may be explained by the induction of an activated microglia phenotype expressing increased levels of TREM2 (triggering receptor expressed on myeloid cells 2) and IDE (insulin degrading enzyme), two microglial proteins that have been demonstrated to regulate Aβ deposition in AD mouse models [12, 14, 48]. A previous study reported that inhibition of calpains mitigates AD pathology and cognitive decline in 3xTgAD mice [30]. We show that L41 has no effect on calpains activity which remains elevated in APP/PS1 mice. Interestingly, selective effect on DYRK1A proteolysis by Leucettine L41 improved synaptic plasticity measured by LTP and rescued spatial learning, working memory and long-term memory impairments in APP/PS1 mice tested with the Y-maze and the Morris water maze tasks at 14 months of age. These findings suggest that preventing DYRK1A proteolysis is sufficient to observe disease-modifying effects in this mouse model. This is supported by comparative evaluation of another synthetic analogue of Leucettamine B, the LeuI in APP/PS1 mice at the same age. As showed in vitro (see Fig. 1b), LeuI is unable to prevent DYRK1A proteolysis. Using similar experimental conditions, we compared LeuI and L41-treated APP/PS1 mice (Additional file 5: Figure 5A-F). No rescue of DYRK1A_*FL*_ levels and DYRK1A_*T*_ in astrocytes or no significant decrease of pro-inflammatory cytokines were observed in LeuI treated animals (Additional file 5: Figure 5A-C). Moreover, no decrease of the amyloid plaque burden and no improvement of the spatial / long-term memory were measured (Additional file 5: Figure 5D-F). These data confirm the role of DYRK1A proteolysis in AD progression and the potential interest of this mechanism as a new therapeutic target to counteract the disease.

## Conclusion

In conclusion, we provide several evidences that DYRK1A is proteolyzed in both AD patients and APP/PS1 mice. We show that truncated forms of DYRK1A accumulate in astrocytes without consequences on its kinase activity. However, kinase specificity of truncated DYRK1A is reduced leading to increase its affinity toward STAT3α, a major regulator of inflammation We demonstrate that decreasing production of DYRK1A truncated forms by Leucettine L41 in an AD-like mouse model, reduces levels of inflammatory cytokines, improves clearance of amyloid-β plaques through microglia recruitment and activation, and consequently improves synaptic plasticity and memory. These data confirm the interest of L41 and eventually other inhibitors of DYRK1A proteolysis for AD.

## Additional files

Additional file 1: Supplementary data figure 1. (JPG 27 kb)

Additional file 2: Supplementary data figure 2. (JPG 137 kb)

Additional file 3: Supplementary data figure 3. (JPG 415 kb)

Additional file 4: Supplementary data figure 4. (JPG 647 kb)

Additional file 5: Supplementary data figure 5. (JPG 186 kb)

Additional file 6: Supplementray Data text. (DOCX 230 kb)
