# Supplementary figures and images for: Inhibition of DYRK1A proteolysis modifies its kinase specificity and rescues Alzheimer phenotype in APP/PS1 mice
Source: Acta Neuropathol Commun. 2019 Mar 18;7:46. doi: 10.1186/s40478-019-0678-6 (PMC6421685; doi:10.1186/s40478-019-0678-6)

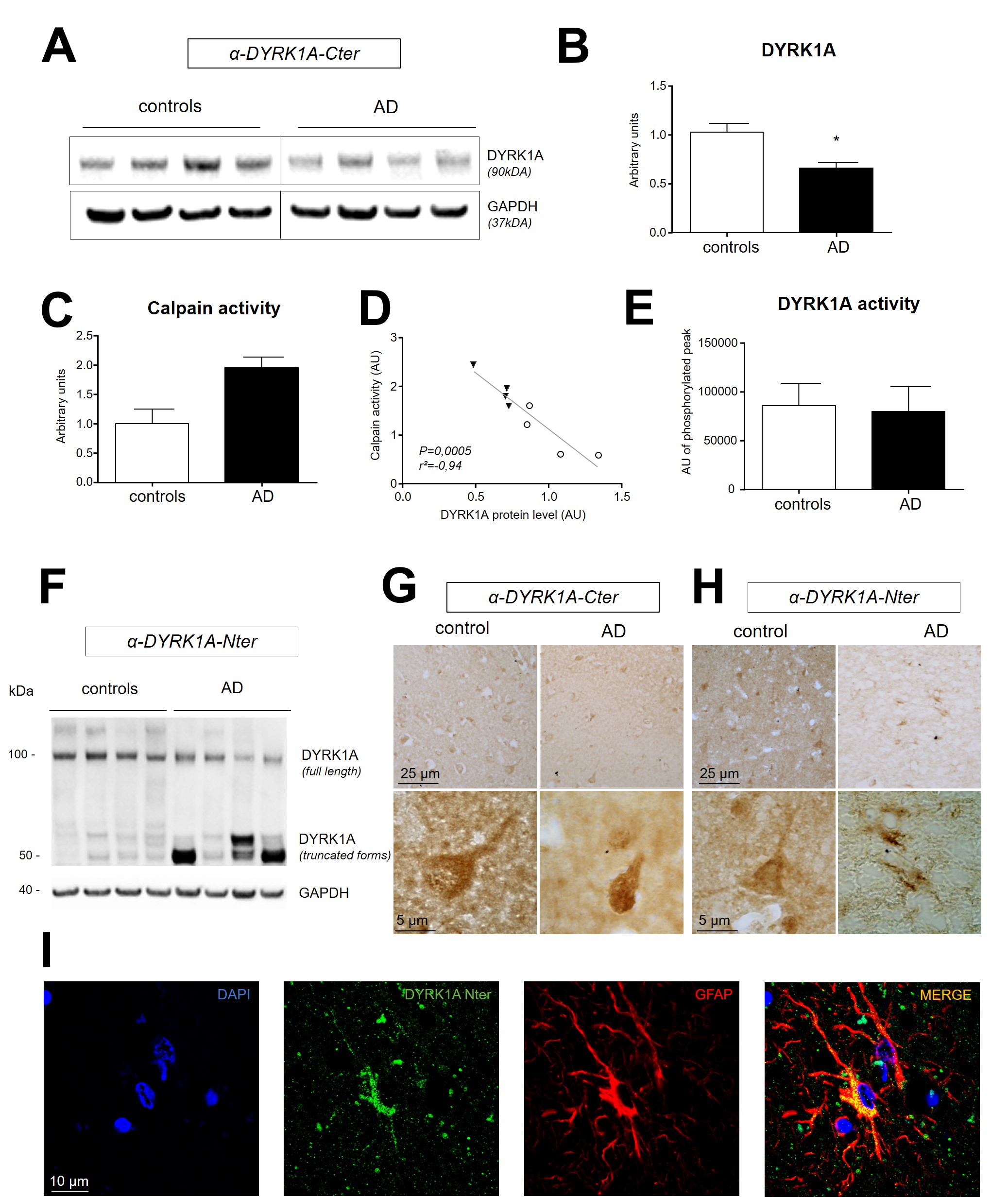

Supplement: Supplementary file 1 — Supplementary data figure 1. (JPG 27 kb) [file 40478_2019_678_MOESM1_ESM.jpg]

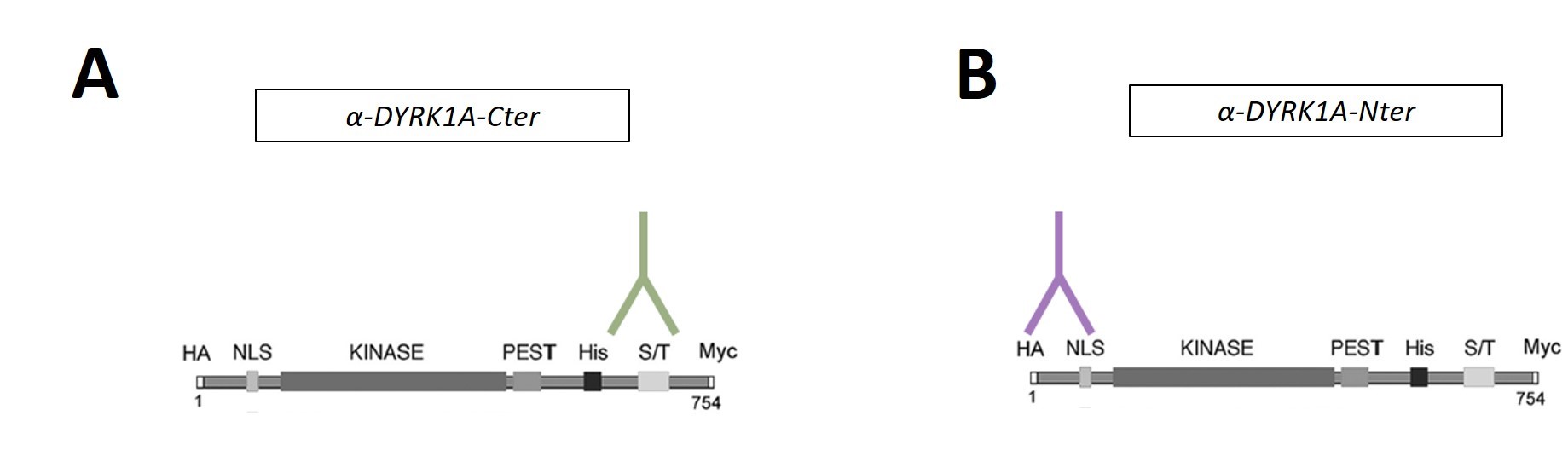

Supplement: Supplementary file 2 — Supplementary data figure 2. (JPG 137 kb) [file 40478_2019_678_MOESM2_ESM.jpg]

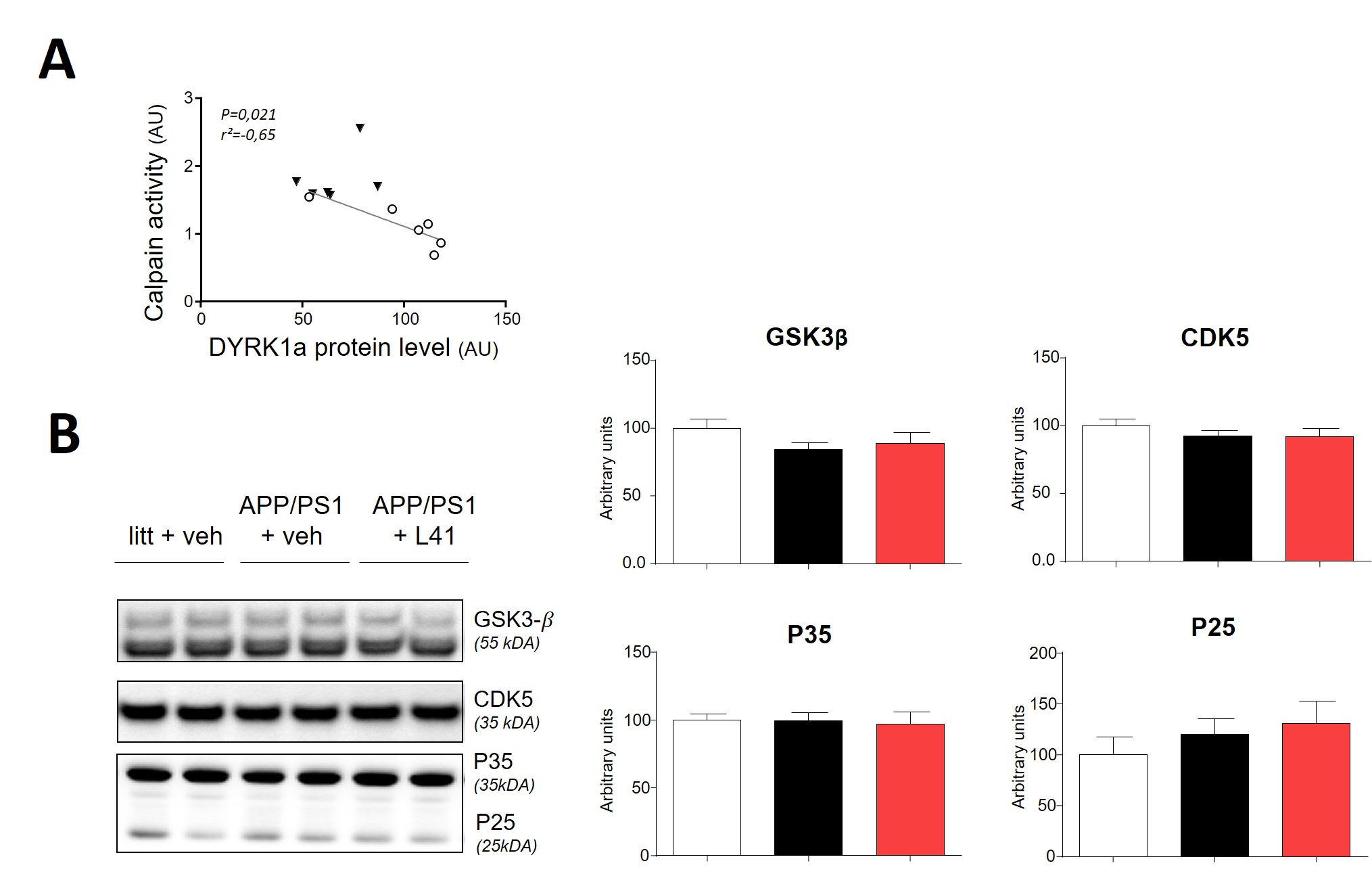

Supplement: Supplementary file 3 — Supplementary data figure 3. (JPG 415 kb) [file 40478_2019_678_MOESM3_ESM.jpg]

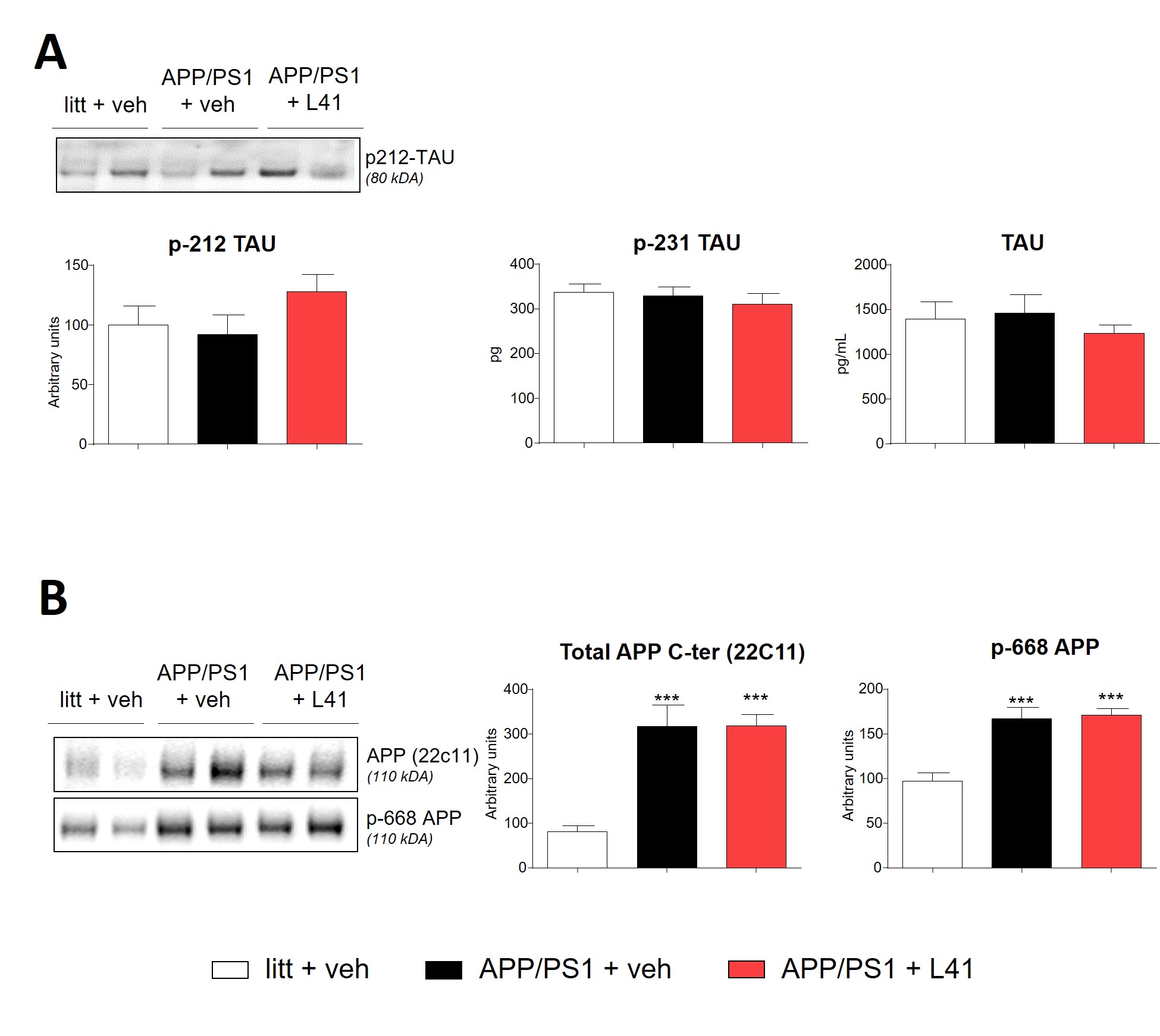

Supplement: Supplementary file 4 — Supplementary data figure 4. (JPG 647 kb) [file 40478_2019_678_MOESM4_ESM.jpg]

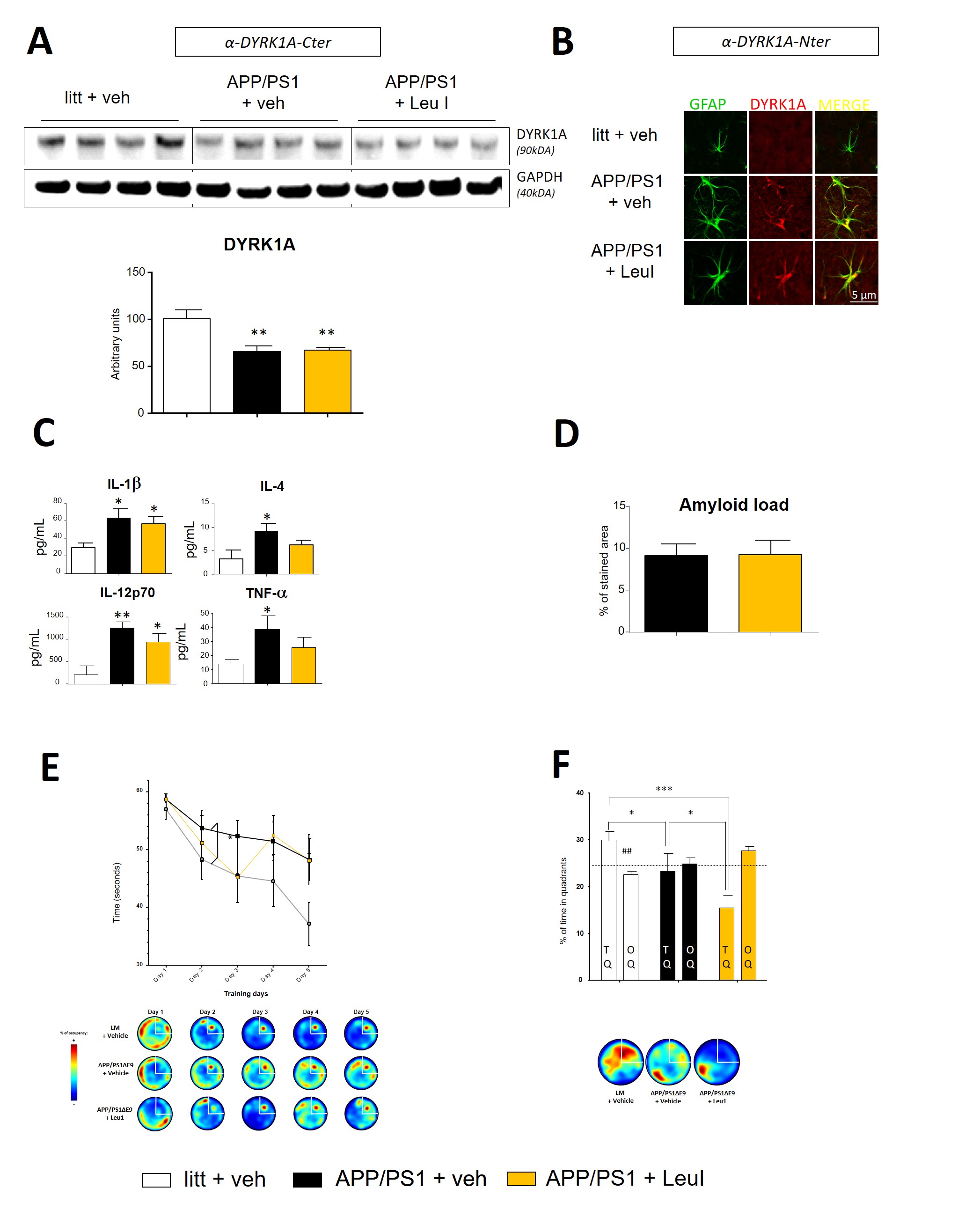

Supplement: Supplementary file 5 — Supplementary data figure 5. (JPG 186 kb) [file 40478_2019_678_MOESM5_ESM.jpg]
