# Supplementary material for: Inhibition of DYRK1A proteolysis modifies its kinase specificity and rescues Alzheimer phenotype in APP/PS1 mice
Source: Acta Neuropathol Commun. 2019 Mar 18;7:46. doi: 10.1186/s40478-019-0678-6 (PMC6421685; doi:10.1186/s40478-019-0678-6)
Supplement: Supplementary file 6 — Supplementray Data text. (DOCX 230 kb) [file 40478_2019_678_MOESM6_ESM.docx]

**Inhibition of DYRK1A proteolysis modifies its kinase specificity and rescues Alzheimer phenotype in APP/PS1 mice**

Benoît Souchet ^1,2 #^, Mickael Audrain ^1^, Jean Marie Billard ^3^, Julien Dairou ^4^, Romain Fol ^1^, Nicola Salvatore Orefice ^1^, Satoru Tada ^1^, Yuchen Gu ^3^, Gaelle Dufayet-Chaffaud ^1^, Emmanuelle Limanton ^5^, François Carreaux ^5^, Jean-Pierre Bazureau ^5^, Sandro Alves ^1^, Laurent Meijer ^6^, Nathalie Janel ^7^, Jérôme Braudeau ^1, 8* #^, and Nathalie Cartier ^1* #^

^1^ INSERM UMR1169, Fontenay-aux-Roses, 92265, France.

^2^ Université Paris Saclay, Saclay, France.

^3^ INSERM UMR894, Centre de Psychiatrie et Neurosciences, Université Paris Descartes, Sorbonne Paris Cité, Paris, France.

^4^ UMR 8601 CNRS, Laboratoire de Chimie et Biochimie Pharmacologiques et Toxicologiques, Université Paris Descartes-Sorbonne Paris Cité, 75270, Paris, France

^5^ Université de Rennes 1, Laboratoire Sciences Chimique de Rennes, UMR CNRS 6226, Groupe ICMV, Rennes, 35042, France.

^6^ ManRos Therapeutics, Hôtel de Recherche, Centre de Perharidy, 29680, Roscoff, France.

^7^ Université Paris-Diderot, Sorbonne Paris Cité, Adaptive Functional Biology, UMR CNRS 8251, Paris, France.

^8^ CEA, DRF Institut François Jacob, MIRCen, Fontenay-aux-Roses, 92265, France

^*^ Indicates joint last authors

^#^ Correspondence should be addressed to Benoit Souchet (benoit.souchet@gmail.com)

**Supplementary Data - Legends**

**Supplementary data 1. Identification of proteolytic processing of DYRK1A in the hippocampus of AD patients.**

(**A**, **B**) Western blot analysis of hippocampus from AD patients (Braak V-VI, Thal IV-V) (n = 4) and healthy controls (n = 4), using the α-DYRK1A-Cter antibody. Results show decreased levels of DYRK1A at 90 kDa (t-test, *p* < 0.05). (**C**) Calpain activity assessed by a fluorescent method show higher levels in hippocampus from AD patients (n = 4) compared to controls (n = 4) (t-test *p* = 0.0571). (**D**) DYRK1A protein levels are negatively correlated with calpain activity (r2 = 0.94, *p* < 0.0005). (**E**) HPLC assay for total endogenous DYRK1A activity, showing no differences between hippocampus from AD patients (n = 4) and controls (n = 4) (t-test, ns). (**F**) Western blot analysis of hippocampus from AD patients (Braak V-VI, Thal IV-V) (n = 4) and healthy controls (n = 4) using the α-DYRK1A-Nter antibody, showing decreased levels of DYRK1A at 90 kDa and increased levels of DYRK1A at 50 kDa. (**G**) Diaminobenzidine (DAB)-staining of hippocampal slices from AD patients and controls using the α-DYRK1A-Cter antibody, showing neuronal staining (see enlargement at the bottom part). (**H**) Diaminobenzidine (DAB)-staining of hippocampal slices using the α-DYRK1A-Nter antibody, showing neuronal and astrocytic staining in controls and AD patients respectively (see enlargement at the bottom part). (**I**) Laser confocal microscopy using DAPI (blue), α-DYRK1A-Nter antibody (green) and anti-GFAP (red) identifying α-DYRK1A-Nter staining in astrocytes of hippocampal slice from AD patients. Data represent the mean $\pm$SEM and were analyzed using Student’s t-test. **p* < 0.05 for the comparison between controls and AD patients.

**Supplementary data 2. DYRK1A-antibodies description**

(**A**, **B**) Diagram of recombinant DYRK1A and epitopes of the DYRK1A antibodies used in this study.

**Supplementary data 3. DYRK1A is negatively correlated by calpain activity whereas GSK3β, CDK5, P35 and P25 are not modified either by L41 treatment**

(**A**) DYRK1A protein levels correlate with calpain activity when vehicle-treated littermates and APP/PS1 mice were analyzed (r2 = 0.65; *p* < 0.05). (**B**) Representative western blot of hippocampus from APP/PS1 mice or littermates treated with vehicle or L41, showing levels of various TAU kinases including GSK3β, CDK5, P35 and P25. All proteins were not modified (One-way ANOVA: 0.3227 ; 0.4868 ; 0.9674 and 0.5952 respectively).

**Supplementary data 4. L41 treatment does not reduce the phosphorylated level of TAU and APP in a DYRK1A specific epitope**

(**A**) Representative western blot of hippocampus from APP/PS1 mice or littermates treated with vehicle or L41, showing levels of phospho-TAU at Thr 212 (p212-TAU). Levels of phosphorylated TAU forms at Thr 212 and Thr 231 (One-way ANOVA: 0.2497 and 0.6609 respectively) as well as total TAU proteins level were not modified (One-way ANOVA: 0.6422).

(**B**) Representative western blot of hippocampus from APP/PS1 mice or littermates treated with vehicle or L41, showing levels of phospho-APP at Thr 668 (p668-APP) which is phosphorylated by DYRK1A and total APP (human + murine protein immunolabeled at C-terminal). Levels of p668-APP and total APP were both higher in vehicle and L41 treated-APP/PS1 compared to littermates (One-way ANOVA, *p* < 0.0005 and *p* < 0.0005 for both protein levels). No difference between vehicle and L41-treated APP/PS1 mice were observed. Data represent the mean ± SEM and were analyzed by one-way ANOVA followed by Tukey’s post hoc. Significant differences between littermates and vehicle-treated APP/PS1 mice are indicated by ****p* < 0.0005. Significant differences between vehicle- and l41-treated APP/PS1 mice are indicated by ###*p* < 0.0005.

**Supplementary data 5: Consequences of no prevention of DYRK1A proteolysis. The LeuI treatment did not restored normal concentration of inflammatory mediators, aggregated amyloid load and behavioral deficits in APP/PS1 mice.**

(**A**) Representative western blot of hippocampus from APP/PS1 mice or littermates treated with vehicle or LeuI, showing lower levels of DYRK1A (90kDa) immunoblotting with the α-DYRK1A-Cter antibody in vehicle-treated APP/PS1 mice (n = 6) than in littermates (n = 6) (One-way ANOVA, *p* < 0.005). DYRK1A protein levels in LeuI-treated APP/PS1 mice (n = 7) were the same than in vehicle-treated APP/PS1 mice (One-way ANOVA, ns). (**B**) Laser confocal microscopy showing double staining using α-DYRK1A-Nter antibody (red) and anti-GFAP (green). α-DYRK1A-Nter staining was localized in the GFAP positive area in vehicle- and LeuI-treated APP/PS1 mice in contrast to littermates. (**C**) The concentrations of inflammatory mediators were no change between vehicle- and LeuI-treated APP/PS1 mice (One-way ANOVA: IL-1$\beta$, IL-4, IL-12p70, ns). (**D**) Laser confocal microscopy showing double staining, using 4G8 antibody (white) and DAPI (blue), of hippocampal slices from vehicle- (n = 6) or LeuI- (n = 6) treated APP/PS1 mice. The surface was covered by plaques in the hippocampi of both mice were similar (t-test, ns). (**E**) Escape latency of vehicle-treated littermate controls or vehicle- or LeuI-treated APP/PS1 mice. The time to reach the platform was different between the groups (2-way ANOVA: Group effect: F2,110=3.68; *p*=0.028; Time effect: F4,110=7.23; p<0.0001; Group x Time interaction: F8,110<1; ns). Vehicle- and LeuI-treated APP/PS1 mice were impaired relative to vehicle-treated littermate controls (*p* = 0.02 and 0.05). (**F**) Probe trial performance at 72h. (Two-way ANOVA, Group effect: F2.46 = 1.315, *p* = ns; quadrant effect: F1.46 = 12.58, *p* = 0.009; Group x quadrant interaction effect: F2.46 = 5.27, *p* = 0.0087). Vehicle- and Leu I-treated APP/PS1 mice were impaired relative to vehicle-treated littermates (*p* = 0.0498 and 0.0014). Target quadrant (TQ) and other quadrants (OQ). Data represent the mean ± SEM and were analyzed by one-way ANOVA followed by Tukey’s post hoc test or Student’s t-test. Significant differences between littermates and vehicle-treated APP/PS1 mice are indicated by **p* < 0.05 and ***p* < 0.005. Significant differences between vehicle- and l41-treated APP/PS1 mice are indicated by #*p* < 0.05 and ##*p* < 0.005.

**Supplementary Data - Material and Methods**

*Human brain samples*

Postmortem samples were obtained from brains collected as part of the Brain Donation Program of the GIE-Neuro-CEB Brain Bank. Autopsies were carried out by accredited pathologists after receiving informed consent from the relatives, in accordance with French Bioethics laws. Hippocampal samples from four patients with sporadic forms of AD (male and female; (Braak V-VI, Thal IV-V); aged between 69 and 89 years, with a postmortem interval (PMI) of 30 to 59 hours) and four age-matched control subjects (male and female, aged between 58 and 89 years, PMI of 6 to 63 hours) were used in this study (See below Table 1). Proteolysis of DYRK1A is not influenced by PMI of 3 hours at 4°C and 25°C according previous study [[23](#_ENREF_23)]. Influence of longer PMI has not been tested. However, the 82 years-healthy man showed a PMI longer than all PMI from Alzheimer’s cases, and may could allow us to rule out the fact that a longer PMI also does not affect DYRK1A proteolysis.

|  |  |  |  |  |  |  |  |
| --- | --- | --- | --- | --- | --- | --- | --- |
|  |  | **Gender** | **Age (yrs)** | **PMI (hrs)** | **Clinical AD status** | **Diagnosis** |  |
|  |  | M | 73 | 54 | Braak V, Thal IV, CAA | AD |  |
|  |  | F | 89 | 59 | Braak VI, Thal V, CAA | AD |  |
|  |  | M | 70 | 30 | Braak VI, Thal V, CAA | AD |  |
|  |  | M | 69 | 30 | Braak VI, Thal V, CAA | AD |  |
|  |  |  |  |  |  |  |  |
|  | Mean | M (75%) | 75,25 | 43,25 | Braak VI, Thal V, CAA | AD |  |
|  |  |  |  |  |  |  |  |
|  |  | M | 58 | 6 | Braak I-II | Ctrl |  |
|  |  | M | 82 | 63 | Braak II, Thal I | Ctrl |  |
|  |  | F | 89 | 12 | / | Ctrl |  |
|  |  | M | 69 | 6 | / | Ctrl |  |
|  |  |  |  |  |  |  |  |
|  | Mean | M (75%) | 74,5 | 21,75 | Braak I, Thal I | Ctrl |  |

*Western blotting*

Similar methods and material were used for this part. However, additional antibodies were used (p-TAU Thr 212 44-740 G (1/1000, Thermofisher), GSK3-β ab73173 (1/500, Abcam), CDK5 EP715Y (1/500, Abcam), P35/P25 C64B10 (1/500, Cell signaling), Total APP (C-ter) clone 22c11 (1/1000, Millipore), p-APP Thr668 SAB4300153 (1/1000, Sigma). Various secondary antibodies were also used (ECL Anti-mouse horseradish peroxidase linked, 1/2000, GE Healthcare; ECL Anti-rabbit horseradishpPeroxidase linked, 1/2000, GE Healthcare). Membranes were developed using enhanced chemiluminescence (Thermo Fisher Scientific). Signals were detected with Fusion FX7 (Vilber Lourmat) and analyzed and quantified using ImageJ (NIH).

*ELISA*

Hyperphosphorylated Tau and total TAU protein were quantified using the Innogenetics Phospho-

the MSD Phospho (Thr231)/Total Tau Kit (Meso Scale Diagnostics). The ELISA was performed according to the kit manufacturer’s instructions in each case.

*Leucettine LeuI treatment*

The pre-weighed compound was dissolved in DMSO/PEG300/water (5/35/60) to a final concentration of 2 mg/mL for a dose of 20 mg/kg. The formulation was prepared on the day of the in vivo experiment. The mice received five intraperitoneal injections per week for four weeks.
